# Supplementary figures and images for: A Genome-wide Association study of Buccal Mucosa Cancer in India and Multi-ancestry Meta-analysis Identifies Novel Risk Loci and Gene-environment Interactions
Source: medRxiv. 2025 Apr 17:2025.04.16.25325815. Preprint. [Version 1] doi: 10.1101/2025.04.16.25325815 (PMC12047951; doi:10.1101/2025.04.16.25325815)

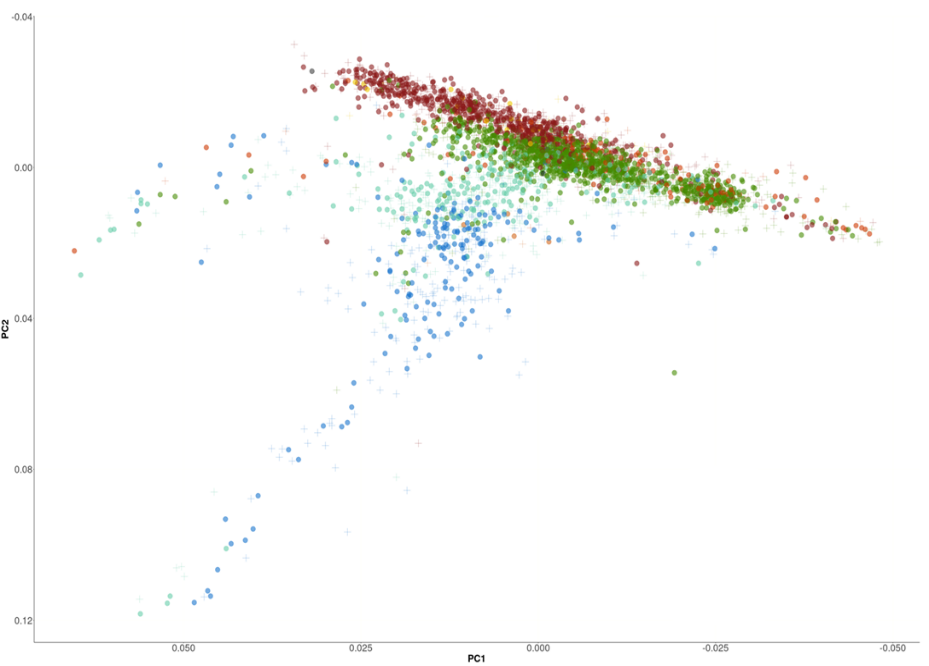

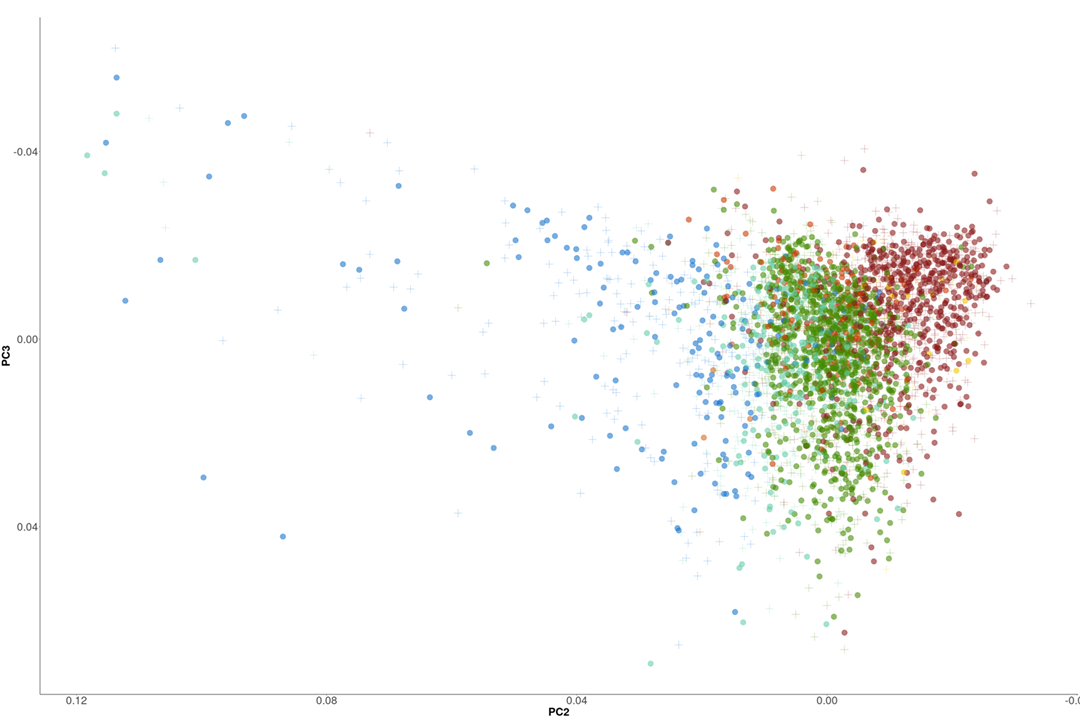

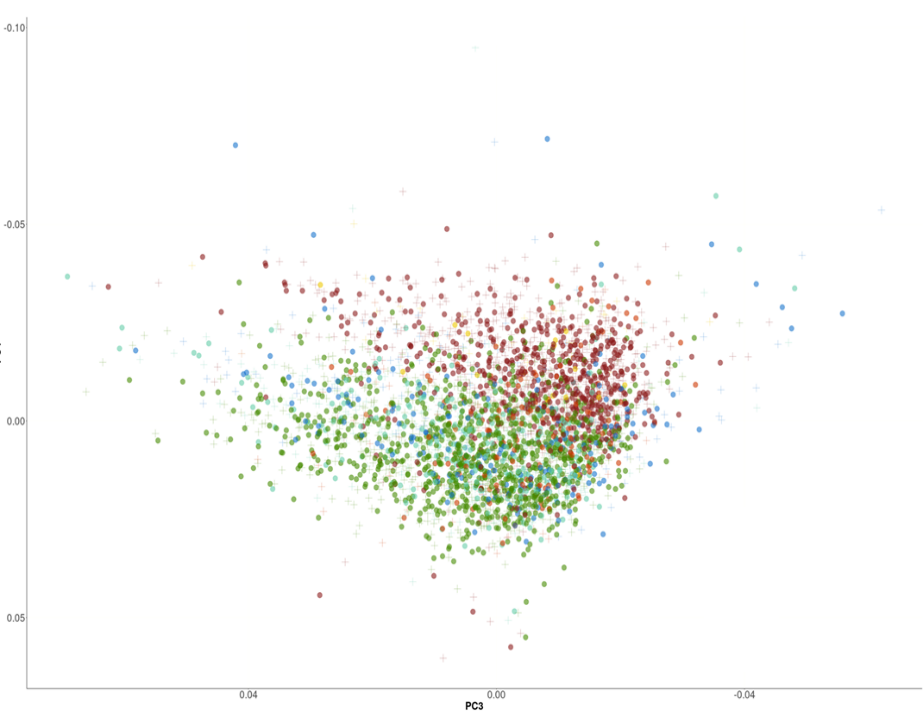

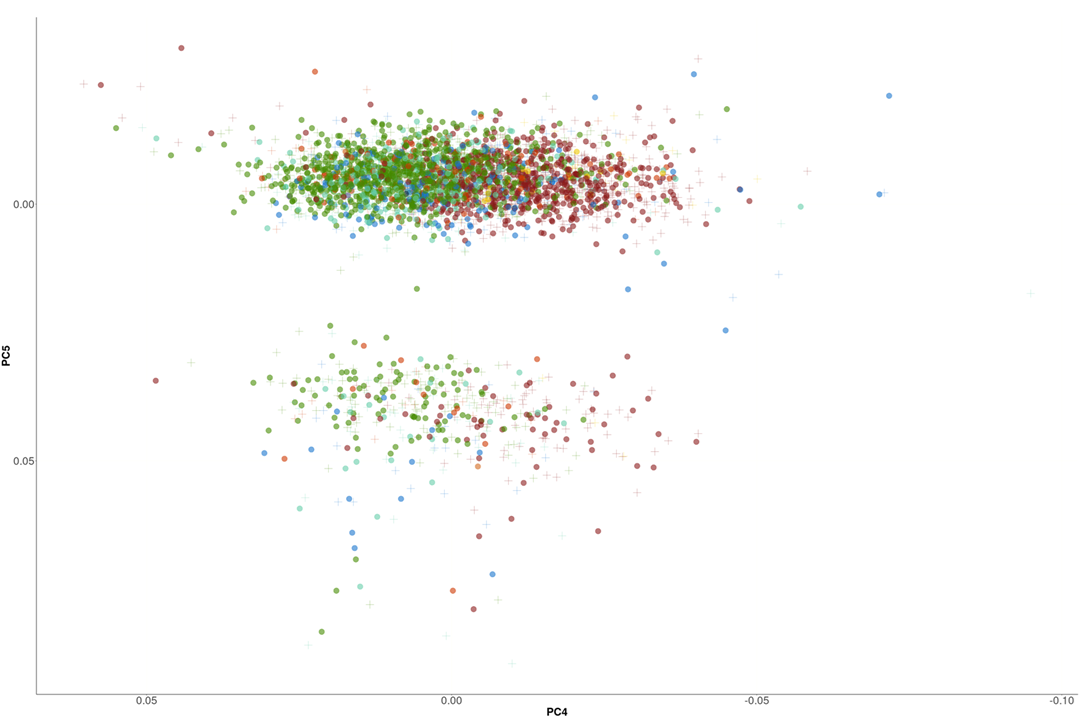

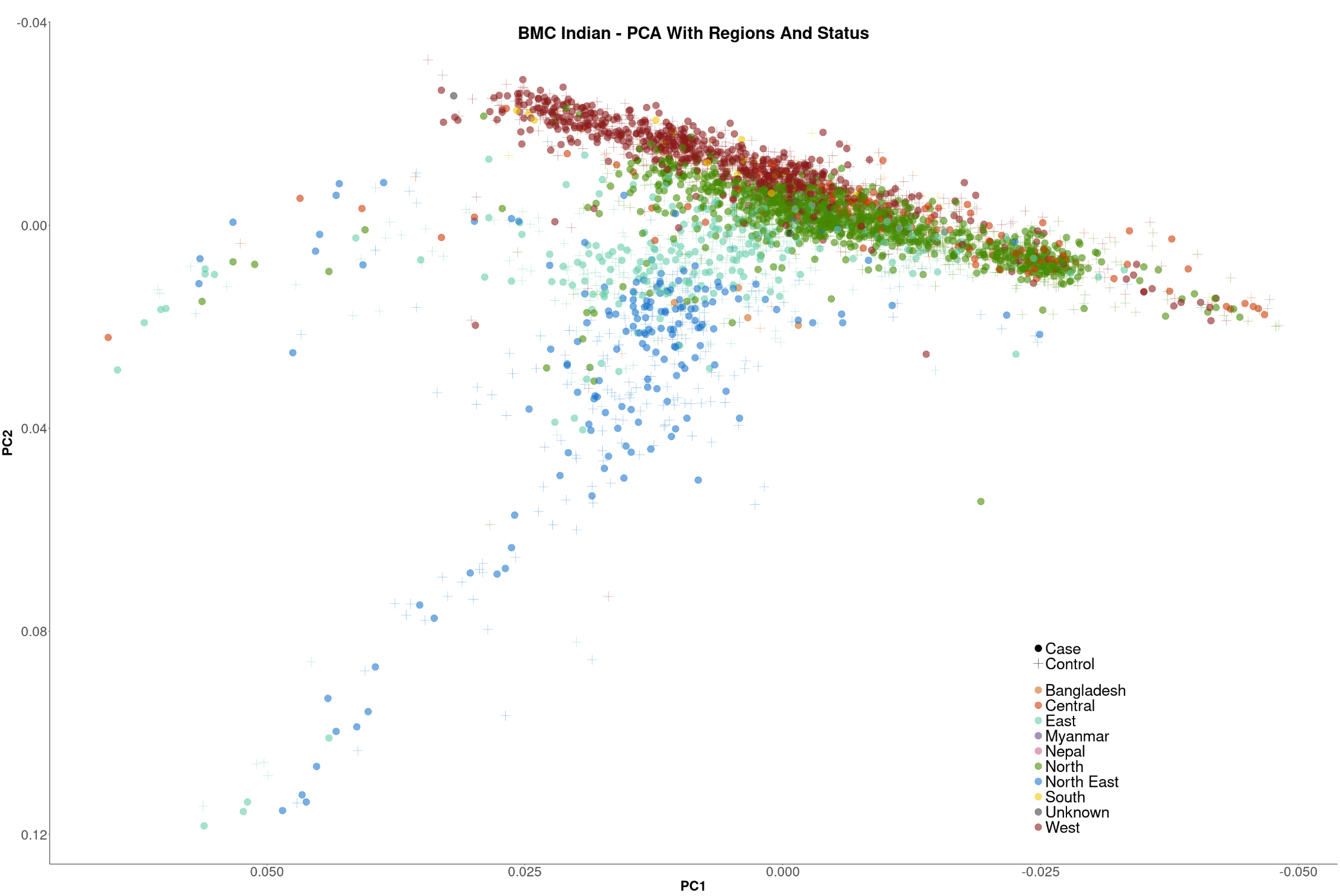

Supplement: Supplement 1 [file media-1.docx]

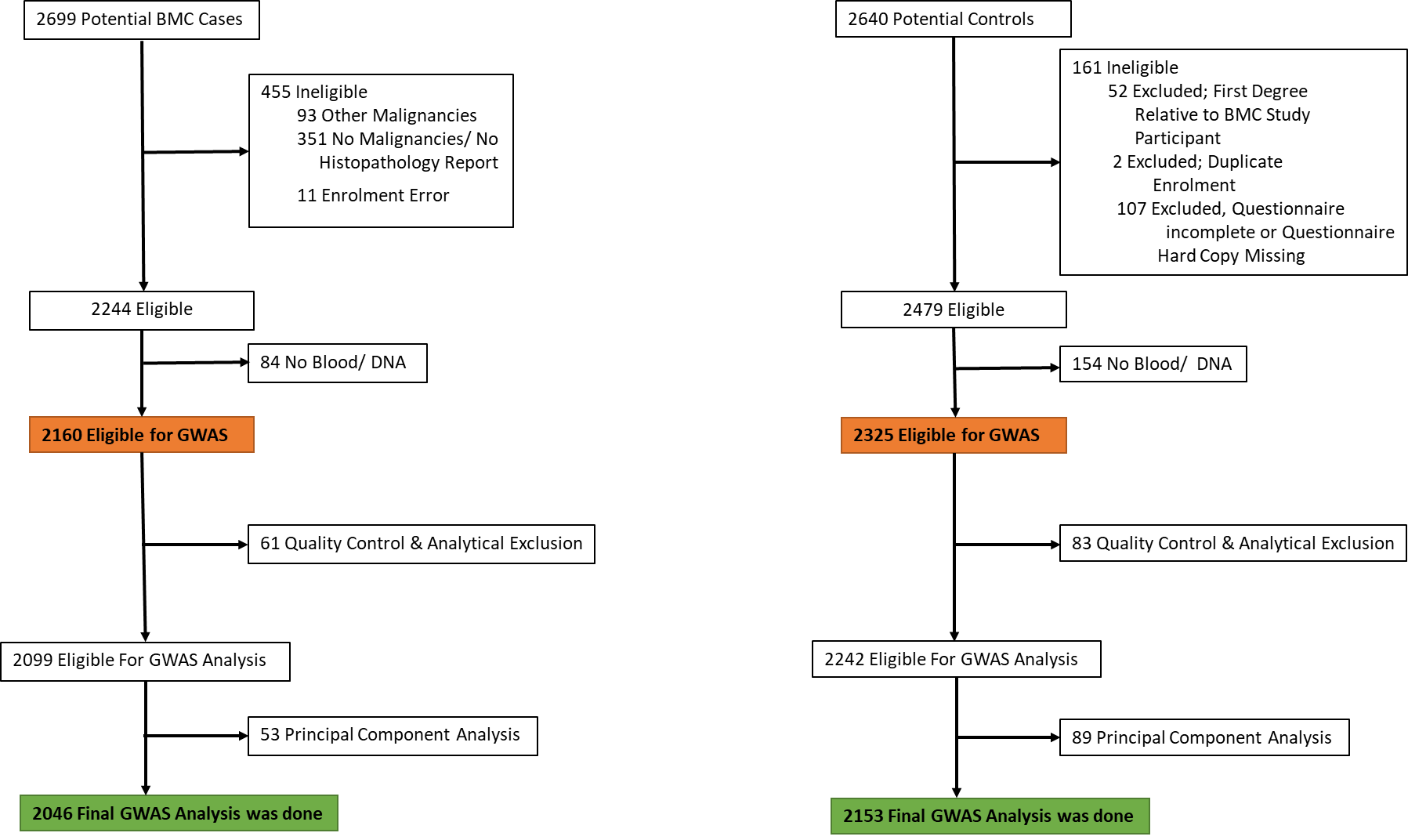

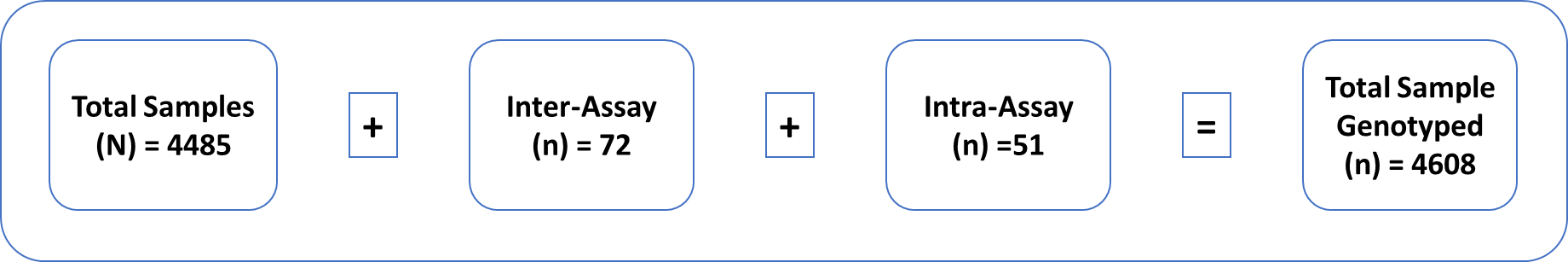

Supplement: Supplement 3 [file media-3.docx]
