# Supplementary material for: A Genome-wide Association study of Buccal Mucosa Cancer in India and Multi-ancestry Meta-analysis Identifies Novel Risk Loci and Gene-environment Interactions
Source: medRxiv. 2025 Apr 17:2025.04.16.25325815. Preprint. [Version 1] doi: 10.1101/2025.04.16.25325815 (PMC12047951; doi:10.1101/2025.04.16.25325815)

**Supplemental Figure 3.** POPCORN analysis estimating genetic correlation across trans-ancestry populations regarding oral cavity cancer risk.


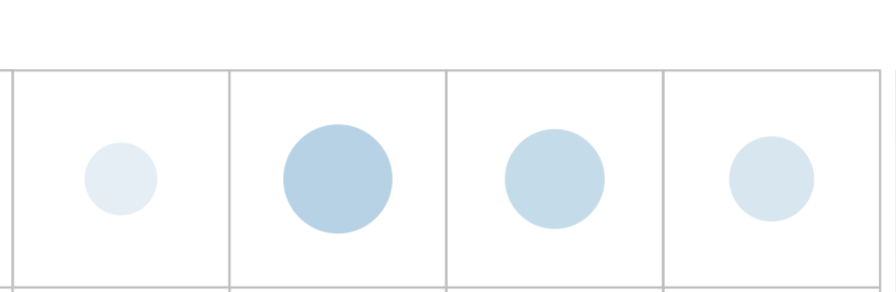


**Europe**

**Hispanic**

**Taiwan**

**North**

**America**

**India**


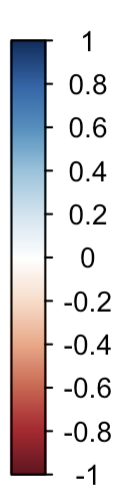

Supplement: Supplement 2 [file media-2.docx]
